# The Relationship Between Noise-Induced Hearing Loss Awareness and the Use of Personal Listening Devices in Makkah Region, Saudi Arabia

**DOI:** 10.7759/cureus.37111

**Published:** 2023-04-04

**Authors:** Faisal Alzahrani, Saad M Alharthi, Sanad M Alharthi, Abdulrahman F Kabli, Abdulrahman Baabdullah, Alwaleed S Alzahrani, Emad Baatiyyah, Abdulaziz F Altowairqi, Shahd Alshareef, Razan M Jan, Abdullah A Khafagy, Mokhtar M Shatla

**Affiliations:** 1 Medicine and Surgery, Umm Al-Qura University, Makkah, SAU; 2 Otorhinolaryngology - Head and Neck Surgery, King Abdulaziz Specialist Hospital, Taif, SAU; 3 Otolaryngology - Head and Neck Surgery, King Fahad General Hospital, Jeddah, SAU; 4 Otolaryngology - Head and Neck Surgery, King Fahad Armed Forces Hospital, Jeddah, SAU; 5 Community Medicine and Pilgrims Healthcare, Umm Al-Qura University, Makkah, SAU; 6 College of Medicine, Umm Al-Qura University, Makkah, SAU

**Keywords:** nihl, volume, noise, knowledge, hearing, awareness, audio devices

## Abstract

Introduction

Noise-induced hearing loss (NIHL) is one of the most common avoidable reasons for hearing impairment worldwide. Work-related, genetic, infectious, and environmental factors all have a part in defining the level of hearing impairment. Nevertheless, the use of personal listening devices (PLDs) is popular nowadays, particularly among younger people. Healthy behaviors are needed to prevent them from developing hearing loss. Our objective is to evaluate the knowledge level of NIHL among the people of Makkah, Saudi Arabia, and understand its association with PLDs.

Methods

A cross-sectional survey was performed in December 2022 by sending an online survey on various social media applications. An electronic Arabic questionnaire with a total of 37 questions was designed to explore the participants' demographic data, history of hearing loss, risk factors, attitudes, and awareness of NIHL.

Results

Almost 22% of the study had mild-to-severe hearing impairment. Hearing issues were especially common among male individuals. A higher incidence of hearing impairments was seen in individuals who were utilizing a sound degree of more than 80%. The causes for NIHL comprised exposure to occupational noise, duration of the listening session per day, and the level to which the sound of the television or the broadcasting was raised. Approximately 77% of the participants preferred to reduce the sound of their personal audio devices (PADs) to prevent NIHL.

Conclusion

According to this study, there is a high prevalence of hearing problems in the Saudi population. Most of the respondents understood the risk factors linked to NIHL. There is a need for more NIHL awareness campaigns to educate the Saudi population and reinforce positive, healthy listening habits.

## Introduction

Hearing loss (HL) affects the ability of people to differentiate between sounds appropriately, making it difficult to understand basic conversations and daily life situations. Also, hearing loss in early childhood can have a considerable negative effect on both development and educational growth, adversely impacting emotional health and social well-being [1]. HL is a critical issue worldwide, affecting 6.1% of the global population [2,3]. Previously published studies have shown that the occurrence rate of HL increased from 15% to nearly 19% in a group of young people aged between 12 and 19 years old [4]. HL can occur at any age, and the reasons for HL vary between adults and children. The causes in children are a first-degree relative history of HL or infection, while in adults, HL is probably due to increased age and prolonged noise exposure [5]. Noise-induced hearing loss (NIHL) happens as a consequence of exposure to loud sounds; these sounds affect the sensory hair cells in the inner ear, which is one of the most common causes of HL [6-8]. The source of loud noise differs and can be either occupational or recreational [9,10]; although noise related to work can be more serious, recreational noises are more frequent in the current period. NIHL has become an international issue in the last two decades due to the growing use of smartphones [6]. Additionally, there has been a rise in the use of personal listening devices (PLDs), which involve headphones and earphones [11,12]. Misuse of these devices can lead to difficulty in understanding speech, tinnitus, unsteadiness, and reduced hearing capability [13]. For this reason, many articles have been written to explore people's beliefs and attitudes regarding NIHL and PLD use. Thus, our survey aims to assess the awareness level of NIHL and the use of PLDs and determine the risk factors, signs, and symptoms associated with HL among the general population in the Makkah region of Saudi Arabia.

## Materials and methods

A cross-sectional survey was performed in December 2022 by distributing an online questionnaire on social media platforms to collect information on participants’ demographics, history of hearing loss, risk factors, beliefs, and knowledge about NIHL as well as to assess society’s awareness of NIHL from PLDs among the general population in the Makkah region of Saudi Arabia, using a validated version of a self-administered questionnaire in the Arabic language [14].

The sample size was decided by using a Raosoft calculator to be more than 380 subjects, with a confidence interval of 95% and a level of significance (P-value) of 5%. The questionnaire was completed by 384 people at random.

We involved only participants from the Makkah region who were ≥18 years old and who agreed to participate in this survey. Partially filled questionnaire submissions were eliminated from the study.

The questionnaire included a total of 37 items distributed into six categories. The initial section consisted of six items to collect personal data. The second section contained five items about medical history. The third section comprised five items about the utilization of PLDs. The fourth section similarly had five items and was designed to evaluate the symptoms of hearing impairment. The fifth section contained a total of 11 items used to assess the knowledge and beliefs regarding NIHL. The last section consisted of five items about the protective measures to stop NIHL.

SPSS Statistics version 22 (IBM Corp., Redmond, WA) was utilized to analyze the data. Categorical variables are represented as frequency (percentage) comprising participants’ demographic data, history of hearing problems, risk factors, knowledge, beliefs, and practices related to hearing problems. Cross-tabulation was used to show the distribution of individuals’ hearing impairment with their different demographic data and described risk factors. The Pearson chi-square test was utilized to evaluate the significance of relationships.

The Biomedical Research Ethics Committee of Umm Al-Qura University approved this study.

## Results

A total of 384 individuals were surveyed: 56.5% were women, 97% were under 50 years old, 90.9% were Saudi, 44.5% had a college degree, 85.2% were nonsmokers, and 91% had no chronic diseases. Of the participants, 77.9% did not exhibit any signs of hearing impairments. However, mild, moderate, and severe hearing loss was found in nearly 13.8%, 5.7%, and 2.6% of the individuals, respectively. Furthermore, a family history of hearing impairments was described by about 51.8% (Table 1).

**Table 1:** Bio-demographic data of the sampled population, Makkah Region, Saudi Arabia

Variables		Frequency	Percent
Age	From 18 to 25	136	35.4
	From 26 to 35	134	34.9
	From 36 to 50	103	26.8
	More than 50	11	2.9
Gender	Male	167	43.5
	Female	217	56.5
Nationality	Saudi	349	90.9
	Non-Saudi	35	9.1
Educational level	Primary or less	5	1.3
	High school level	111	28.9
	University level	171	44.5
	Postgraduate level	97	25.3
Smoking	Yes	57	14.8
	No	327	85.2
Chronic health problems	Diabetes	18	4.7
	Hypertension	12	3.1
	Cardiac disease	4	1.0
	I don’t have chronic disease	350	91.1
Do you have any signs of hearing problems	Mild	53	13.8
	Moderate	22	5.7
	Severe	10	2.6
	None	299	77.9
Family history of hearing problems	Yes	199	51.8
	No	185	48.2

The risk factors for NIHL involved work-related noise (40.4%), choice of using earphones (63.5%), and a high frequency of sessions (6 to ≥10; 62.2%) where the individual was exposed to a loud noise sound corresponding to the following parameters: >3 h per session (31%) and a sound degree in the range of 80%-100% (23.4%). Forty-nine percent of the study participants engaged in more than 10 sessions weekly where they would listen to a noise sound for <1 h/day (Table 2). A considerable number of the individuals in this study also suffered tinnitus (44.5%), whereas others described that people occasionally complained that they spoke too noisily (38%). Others reported the occasional need to raise the sound of the television or radio (53.6%). This study found that 89.3% of participants needed one hour to adjust to the level of loudness produced by the noise sound (Table 3).

**Table 2:** Distribution of risk factors related to noise-induced hearing loss. How often are the people surrounding me affected by the noise from my PAD? PAD: personal audio device

Variables		Frequency	Percent
Exposure to occupational noise	Yes	155	40.4
	No	229	59.6
Preferred type of audio device	External PADs	35	9.1
	Earphones	244	63.5
	Car PADs	67	17.4
	Headphones	38	9.9
Number of hearing sessions per week	More than 10	189	49.2
	6 - 9	50	13
	1 - 5	87	22.7
	Never	58	15.1
Duration of the listening session per day (h)	Less than 1	166	43.2
	1 - 2	99	25.8
	3 - 5	71	18.5
	More than 5	48	12.5
How often are the people surrounding me affected by the noise from my PAD	Never	259	67.4
	Sometimes	102	26.6
	Usually	16	4.2
	Always	7	1.8
Typical level of volume used (%)	0 - 49	135	35.2
	50 - 69	87	22.7
	70 - 79	72	18.8
	80 - 89	40	10.4
	90 – 100	50	13

**Table 3:** Signs and symptoms associated with noise-induced hearing loss in Makkah Region, Saudi Arabia

Variables		Frequency	Percent
Ringing in the ears	Never	178	46.4
	Sometimes	171	44.5
	Usually	15	3.9
	Always	20	5.2
People said I talk loud	Never	143	37.2
	Sometimes	146	38
	Usually	41	10.7
	Always	54	14.1
I tend to ask “What ?” repeatedly in a conversation	Never	101	26.3
	Sometimes	200	52.1
	Usually	52	13.5
	Always	31	8.1
Increasing the volume of the TV or radio is something I do	Never	85	22.1
	Sometimes	206	53.6
	Usually	50	13.0
	Always	43	11.2
Time I need to adapt to surrounding environmental sound when exposed to loudness (h)	1	343	89.3
	5	33	8.6
	10	2	.5
	15	6	1.6

An assessment of individual beliefs regarding the risk factors of NIHL showed fascinating findings. Most of the individuals in this study were aware that high sound degrees could lead to hearing impairments (87.2%) and that staying in a noisy setting could also adversely impact hearing (83.9%). They also identified that a prior hearing problem could get worse by sustained exposure to loud sound (79.4%) (Table 4). Furthermore, about 48% of the study individuals assumed that daily dialog becoming harder to follow was a sign of hearing loss. Likewise, 26.6% of the study individuals believed that tinnitus could also be a sign of hearing loss. Curiously, 70.6% of the individuals in this study understood that hearing impairments caused by noise are avoidable, and almost half of them believed that they had adequate knowledge about the risks of loud sounds on their hearing capability. Yet, a considerable number of study individuals did not understand the minimum duration (35.2%) or the minimum level (45.3%) that can adversely affect hearing capability (Table 4).

**Table 4:** Knowledge and beliefs about noise-induced hearing loss

		Frequency	Percent
Do high volume levels affect hearing?	Yes	335	87.2
	No	17	4.4
	I don’t know	32	8.3
Does living or working in a noisy environment affect hearing?	Yes	322	83.9
	No	22	5.7
	I don’t know	40	10.4
Hearing impairment could get worse by listening to loud sounds	Yes	305	79.4
	No	22	5.7
	I don’t know	57	14.8
Does the hearing of low/muffled voices during daily conversation indicate the early signs of hearing impairment?	Yes	185	48.2
	No	93	24.2
	I don’t know	106	27.6
Is the sensation of ringing in the ear a sign of hearing impairment?	Yes	102	26.6
	No	98	25.5
	I don’t know	184	47.9
Does the frequent increase in TV/radio volume indicate a sign of hearing impairment?	Yes	210	54.7
	No	91	23.7
	I don’t know	83	21.6
Are noise-induced hearing problems preventable?	Yes	271	70.6
	No	22	5.7
	I don’t know	91	23.7
Do I currently have enough information concerning the danger posed by exposure to loud noises on hearing ability?	Yes	147	38.3
	No	160	41.7
	I don’t know	77	20.1
The minimum duration of listening to a loud noise source that could negatively affect one’s hearing is	30 min	113	29.4
	1 h	56	14.6
	1 and half h	20	5.2
	2 h or more	60	15.6
	I don’t know	135	35.2
The minimum volume level that could negatively affect hearing is (%)	20-40	47	12.2
	41-60	27	7
	61-80	50	13
	81-90	52	13.5
	91-100	34	8.9
	I don’t know	174	45.3

The attitudes and practices correlated to NIHL suggest that a significant number of the study individuals (74.6%) favored decreasing the level of several audio devices as a preventive plan, and a considerable number (81%) suggested that the manufacturer must set sound-warning features on devices (Table 5). Furthermore, most of the study individuals (92.7%) advised fixing an alarm feature in devices to restrict the sound level, whereas 15.9% favored the use of a system to limit sound output. Approximately 35.2% said that their source of information about NIHL was hospitals.

**Table 5:** Practices and attitudes toward noise-induced hearing loss NIHL: noise-induced hearing loss

Variables		Frequency	Percent
Typically accessed source of information about NIHL	Educational campaigns	60	15.6
	Commercial centers	11	2.9
	Schools and job settings	38	9.9
	Hospitals	135	35.2
	Social media	114	29.7
	TV	26	6.8
Do I prefer to decrease the volume of my device over the total time of listening?	Yes	294	76.6
	No	90	23.4
I recommend that the factory should install a voice-limiting feature on my PAD	Yes	311	81
	No	37	9.6
	I don’t know	36	9.4
I’m ready to change my behavior if I hear or see evidence that suggests that loud noise/sound levels affect hearing	Never	198	51.6
	Sometimes	62	16.1
	Usually	101	26.3
	Always	23	6
I recommend putting warning indicators on audio devices to limit volume levels	Yes	356	92.7
	No	28	7.3
I prefer using a program to limit sound levels for me and my family	Never	208	54.2
	Sometimes	50	13.0
	Usually	65	16.9
	Always	61	15.9

Subgroup evaluations relating to the recognition of independent variables implied that older age was associated with a higher number of hearing issues, though this association was not statistically significant (P = 0.063). A higher percentage of males (26.3%) reported experiencing hearing problems as compared to females (18.9%). However, this was not statistically significant (P = 0.081). Additionally, the study did not find any statistically significant associations between educational level, smoking, chronic diseases, and family history (Table 6). The risk factors associated with NIHL included exposure to occupational noise (P = 0.000), increased sound degrees originating from a television or radio (P = 0.000), and >5 h of being exposed to a noise sound (P = 0.013) (Table 7).

**Table 6:** The associations between the reporting of hearing impairment and certain independent variables

Variables		Have hearing impairment		
		Yes, n (%)	No, n (%)	p-value
Gender	Male	44 (26.3)	123 (73.7)	0.081
	Female	41 (18.9)	176 (81.1)	
Age	From 18 to 25	28 (20.6)	108 (79.4)	0.063
	From 26 to 35	27 (20.1)	107 (79.9)	
	From 36 to 50	24 (23.3)	79(76.7)	
	More than 50	6 (54.5)	5 (45.5)	
Educational level	Primary or less	0 (0.0)	5 (100)	0.106
	High school level	32 (28.8)	79 (71.2)	
	University level	37 (21.6)	134 (78.4)	
	Postgraduate level	16 (16.5)	81 (83.5)	
Do you smoke	Yes	18 (31.6)	39 (68.4)	0.063
	No	67 (20.5)	260 (79.5)	
Do you have any of these diseases	Diabetes	6 (33.3)	12 (66.7)	0.151
	Hypertension	5 (41.7)	7 (58.3)	
	Cardiac diseases	0 (0.0)	4 (100)	
	I don’t have a chronic disease	74 (21.1)	276 (78.9)	
Do you know anyone in your family who is diagnosed with hearing problems	Yes	47 (23.6)	152 (76.4)	0.468
	No	38 (20.5)	147 (79.5)	

**Table 7:** The significant associations between the reporting of hearing impairment and certain risk factors and the duration of the listening session per day PAD: personal audio device

Variables		Have hearing impairment		
		Yes, n (%)	No, n (%)	p-value
Exposure to occupational noise	Yes	49 (31.6)	106 (68.4)	0.000
	No	36 (15.7)	193 (84.3)	
Number of hearing sessions per week	+10	37 (19.6)	152 (80.4)	0.541
	6-9	13 (26.0)	37 (74.0)	
	1-5	23 (26.4)	64 (73.6)	
	Never	12 (20.7)	46 (79.3)	
Duration of the listening session per day	<1	29 (17.5)	137 (82.5)	0.013
	1-2	18 (18.2)	81 (81.8)	
	3-5	25 (35.2)	46 (64.8)	
	>5	13 (27.1)	35 (72.9)	
Typical level of volume used	0-49	21 (15.6)	114 (84.4)	0.028
	50-69	23 (26.4)	64 (73.6)	
	70-79	12 (16.7)	60 (83.3)	
	80-89	13 (32.5)	27 (67.5)	
	90-100	16 (32.0)	34 (68.0)	
Increasing The volume Of the TV or radio is something	Never	11 (12.9)	74 (87.1)	0.000
	Sometimes	32 (15.5)	174 (84.5)	
	Usually	21 (42.0)	29 (58.0)	
	always	21 (48.8)	22 (51.2)	
How often are the people surrounding me affected by the noise from my PAD	Never	45 (17.4)	214 (82.6)	0.002
	Sometimes	29 (28.4)	73 (71.6)	
	Usually	8 (50.0)	8 (50.0)	
	Always	3 (42.9)	4 (57.1)	

## Discussion

The negative impacts on human well-being due to loud sound exposure are not instantly perceivable [15]. This study assessed the hearing impairment awareness among the individuals of Makkah, Saudi Arabia, and its association with PLDs.

Those who describe symptoms of hearing issues, such as tinnitus and sound sensitivity, are more likely to accept protective hearing behaviors [16,17]. There is evidence of increased knowledge among adults about the danger of NIHL. Nevertheless, people may be unaware that they are at risk and hence deem it pointless to modify their listening behaviors [18].

A study performed previously revealed that 60.6% of participants used media devices with high-sound settings on a daily basis [19]. In agreement with our study, 49.2% of the participants reported more than 10 hearing sessions weekly. An additional difference is that only 35.2% of the individuals in our study were hearing a sound of less than 50%. This result does not necessarily suggest that the Saudi participants had better listening habits. Approximately 54% of the individuals in our study stated ringing in the ears as compared to 21% of the participants in a study done in Jordan [19].

In the current study, 22.1% of the participants complained of hearing loss. These results were higher than the results described in a previous study [10], where nearly 10% of the participants stated a history of hearing loss. Another study showed a lower prevalence of hearing impairment than the participants who took place in this survey. The study revealed that only 7.3% of the participants reported hearing loss, whereas most of them showed mild hearing impairment only [19,20].

In the present survey, 40.4% of the subjects were exposed to work-related noise. In contrast, a study published in Saudi Arabia revealed that 16.9% of the participants were exposed to occupational noise [21].

In this study, 63.5% chose to use earphones. A comparable finding has been noted in a previous study, where they were utilized by 51% [20]. In the current study, 43.2% of the individuals listened to a noise-emitting sound for <1 h/session. This agrees with a study carried out in Saudi Arabia [21]. In contrast, a higher mean listening time was documented in Malaysia (1.5-3.2 h) [22]. Social and spiritual aspects may have influenced the shorter durations of usage of personal audio devices (PADs) in Saudi society.

Of the participants, 26.6% stated that sometimes the sound of their device was high enough to be heard by the individuals around them. This number is lower than that of the Saudi Arabian study, where 41% of participants reported a similar experience [21].

Our study noticed that more than two-thirds of the participants reported difficulty in listening to others, which is a finding higher than described in the previous study [21].

Of the participants, 87.2% understood that high sound degrees could harmfully impact their hearing. This is in agreement with the survey carried out in Saudi Arabia [21].

This study has some limitations. First, the cross-sectional study proves only association. Second, the limit to a particular region might affect the generalization of the findings. Third, the lack of a standardized questionnaire about the attitudes and knowledge related to NIHL could have limited the comparability of the study results. Fourth, linear regression analysis was not performed.

## Conclusions

The present study has recognized that there is a high prevalence of hearing loss in Saudi society. Most of the individuals were aware that such hearing issues were avoidable. However, many of them were unaware of the minimum duration of exposure to a noise or sound or the sound degree that could lead to hearing damage. The positive attitude of the individuals regarding modifying their lifestyle suggests that there is a need for NIHL awareness campaigns to increase the educational level of society. Our recommendation for future studies is to explore the role of occupation in NIHL.
